# Pharmacoeconomic Analysis of Fixed-Dose Combinations of Proton Pump Inhibitors Available in India

**DOI:** 10.7759/cureus.36112

**Published:** 2023-03-14

**Authors:** Pradnya Deolekar, Kavita Vivek, Souvik Ghosh, Azra Naseem, Mayakalyani Srivathsan, Vivek S Rai, Sonal Signapurkar, Pramila Yadav

**Affiliations:** 1 Pharmacology, D. Y. Patil Deemed to be University, Navi Mumbai, IND; 2 Critical Care, D. Y. Patil Hospital, Navi Mumbai, IND

**Keywords:** pharmaceutical company, prescriptions, health economics, pharmacoeconomics, proton pump inhibitors

## Abstract

Introduction

The global proton pump inhibitors (PPIs) market was valued at US$ 2.9 billion in 2020 and is expected to exhibit a compound aggregated growth rate of 4.30% during the forecast period (2020-2027), as they are regularly prescribed for many gastrointestinal disorders, and the treatment usually lasts for a longer period. PPIs are usually combined with antiemetics and prokinetic drugs. The price of PPIs for the same combination varies a lot, which can lead to a lot of financial burden on the patients.

Objective

To evaluate the cost ratio and percentage cost variation of commonly used PPIs in various combinations.

Methodology

The cost of different brands of commonly used PPIs in combination with other drugs was analyzed in our study. A total of 21 different combinations (10 capsules/tablets for oral use) were tabulated by referring to the “Monthly Index of Medical Specialities” October-December 2021, and 1mg online pharmacy. The cost ratio and percentage cost variation for various brands of a particular strength and dosage form were calculated and compared. Cost ratio > 2 and cost variation > 100% were considered significant.

Results

The results show a huge variation (1788.88%) in costs of different brands with the highest being rabeprazole 20 mg and domperidone 10 mg (cost ratio: 18.88, percentage cost variation: 1788.88%) in oral formulation, followed by pantoprazole 40 mg and itopride 150 mg. The minimum cost ratio (1.35) and percentage cost variation (1.35%) is for pantoprazole 40 mg and levosulpiride 75 mg. Logistic regression analysis between the number of brands and percentage cost variation gives an R^2^ value of 0.0923.

Conclusion

There is a wide variation in the prices of PPIs available in the market, which can inadvertently increase the financial burden of therapy on patients. Physicians need to be made aware of these price differences so that they can choose the best available alternative for patients, which can help in increasing compliance with the prescribed drugs.

## Introduction

Out of all the medicines that have been used to suppress gastric acid production, proton pump inhibitors (PPIs) are the most potent [1]. The primary target for this group of drugs is the H^+^-K^+^ ATPase pump present in the parietal cells of the stomach. The drug gets concentrated in the secretory canaliculus of the parietal cells because of its weak basic nature. It also covalently binds with the ATPase accessible at the luminal surface, because of which the inhibitory effects last much longer than the plasma half-life [2].

PPIs have become the drug of choice for a variety of acid-peptic disorders like peptic ulcer, gastroesophageal reflux disease, gastritis, esophagitis, Zollinger-Ellison syndrome, risk reduction of gastric ulcer associated with non-steroidal anti-inflammatory drugs (NSAIDs), and *Helicobacter pylori* eradication to reduce the risk of duodenal ulcer recurrence in combination with antibiotics. PPIs are also used as a protective agent for stress ulcer disease [3]. Due to its efficient reduction of acid secretion with very minimal side effects, this class of drug has become extremely popular and its use has been widespread.

The Food and Drug Administration defines a combination product as "a product composed of any combination of a drug and a device or a biological product and a device or a drug and a biological product or a drug, device, and a biological product." Fixed-dose combinations (FDCs) are basically two or more combination drugs in a single dosage form [4]. They provide the benefit of increased efficacy with lesser side effects [5]. Practitioners prefer to use this more frequently as it improves patients' compliance with the medications [6]. Better compliance is one of the most important factors guiding the physician's choice of a particular drug, especially in chronic conditions. FDCs also contribute to bringing the overall cost of therapy lower than taking individual drugs.

There is a significant difference in the cost between different brands of the same combination available in the market owing to the competition between them, the market share they have, and the target population. The higher cost adds to the economic burden on patients, which can decrease the compliance of patients to these drugs. According to a report published by Technavio, the PPI drug market is growing at a rate of 4.17% per year between 2020 and 2025 and is expected to have a value of $10.24 billion by 2026. All of this further contributes to the price variation between various brands of PPI in combination with other drugs.

With this background, we decided to start by collecting all the relevant information about various combinations of PPIs available in the market and evaluate the cost to find the ratio between the minimum and maximum value and calculate the percentage cost variation between the brands.

## Materials and methods

The cost of FDCs of PPI drugs in the same strength and oral dosage form (tablets/capsules) available in India was obtained from the “Monthly Index of Medical Specialities" (issued: October-December 2021) and 1mg online pharmacy, as they are a readily available source of drug information and are updated regularly. No generics were included in our study.

The cost of 10 tablets/capsules was recorded. The difference between the maximum and minimum cost of the same drug combination being manufactured by different pharmaceutical companies was calculated.

The cost ratio between the maximum and minimum cost of the same drug combination manufactured by different pharmaceutical companies was calculated as follows:

Cost ratio = maximum cost/minimum cost.

Percentage cost variation was calculated as follows:

Percentage cost variation = maximum cost - minimum cost x 100/minimum cost.

A cost ratio of more than 2 and a percentage cost variation of more than 100% are considered significant and show a high variation in the cost between different brands of the combination.

Statistical analysis

The results from our study were expressed as numbers and percentages. Cost ratio and percentage cost variation were calculated. Graphical representation as per requirement was done. Logistic regression between the number of brands and percentage cost variation was conducted to analyze the correlation.

## Results

A total of 21 different combinations were analyzed for our study. Out of these, 13 were a combination of PPI with anti-emetic drugs, five with anti-motility drugs, two with NSAIDs, and one with antimicrobials.

Table 1 presents details about the cost of FDC with PPI drugs that are available in the market.

**Table 1:** Cost of fixed-dose combinations of proton-pump inhibitors available in the Indian market FDC: fixed-dose combination; IR: immediate release; SR: sustained release; mg: milligrams; INR: Indian rupee.

FDC	Doses (mg)	No. of brands	Minimum cost (INR)	Maximum cost (INR)
Pantoprazole & domperidone	20 + 10	143	44.00	153.00
Pantoprazole & domperidone	40 + 10	1310	54.70	193.22
Pantoprazole & domperidone	40 + 30	1796	46.67	276.50
Pantoprazole & domperidone (IR) + domperidone (SR)	40 + 10 + 20	2	85.50	105.00
Pantoprazole & itopride	40 + 150	40	52.75	384.60
Pantoprazole + levosulpiride	20 + 75	4	140	189
Omeprazole + domperidone	20 + 10	10	45	92.26
Omeprazole + domperidone	20 + 30	100	41	160.6
Omeprazole + amoxicillin + tinidazole (kit)	20 + 750 + 500	9	4.1	25
Rabeprazole + domperidone	20 + 10	563	45	850
Rabeprazole + domperidone	20 + 30	38	32	200.70
Rabeprazole + domperidone (IR) + domperidone (SR)	20 + 10 + 20	1		145
Rabeprazole + itopride	20 + 150	10	100	273
Rabeprazole + levosulpiride	20 + 75	19	96	331
Rabeprazole + diclofenac	20 + 100	10	38	135
Rabeprazole + aceclofenac	20 + 200	10	70	143
Lansoprazole + domperidone	30 + 10	30	39	115
Esomeprazole + domperidone	20 + 30	6	64	106
Esomeprazole + domperidone	40 + 30	419	43.3	209.2
Esomeprazole + levosulpiride	40 + 75	69	160	287.2
Ilaprazole + domperidone	10 + 30	4	108	173.2

**Figure 1:**
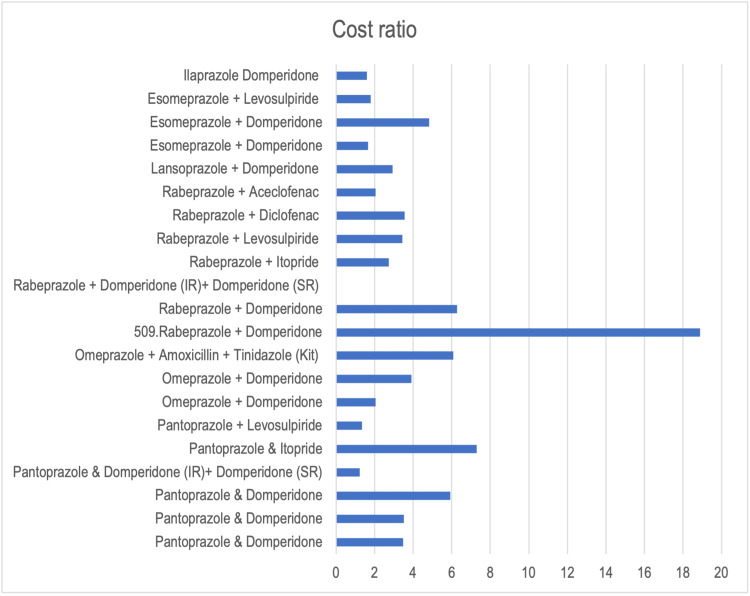
Cost ratio of fixed-dose combinations of proton pump inhibitors IR: immediate release; SR: sustained release.

**Figure 2:**
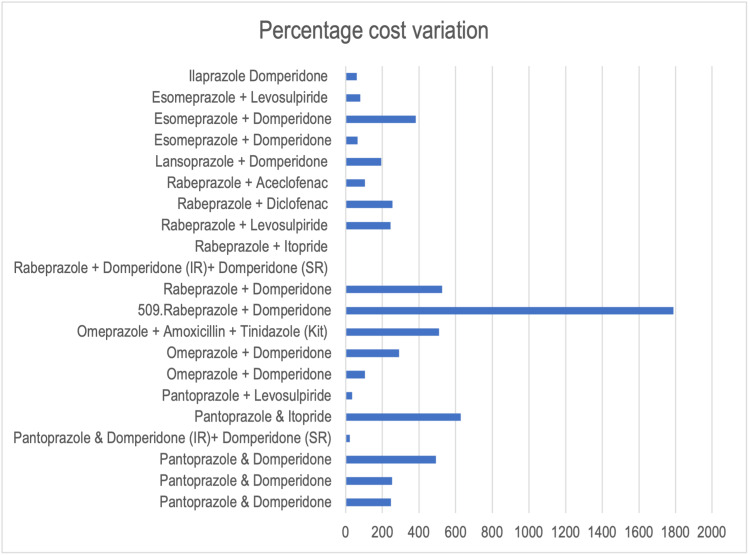
Percentage cost ratio of fixed-dose combinations IR: immediate release; SR: sustained release.

Figures 1, 2 show graphically the cost ratio and percentage cost variation of the FDCs.

Tables 2-4 show regression analysis to check the correlation between percentage cost variation and the number of brands manufacturing a particular combination.

**Table 2:** Summary output of regression analysis

Regression statistics	
Multiple R	0.302848828
R square	0.091717412
Adjusted R square	0.041257269
Standard error	387.2006439
Observations	20

**Table 3:** Analysis of variance df: degree of freedom; SS: sum of square; MS: mean square.

ANOVA					
	df	SS	MS	F	Significance F
Regression	1	272505.6127	272505.6127	1.817620909	0.194318836
Residual	18	2698638.095	149924.3386		
Total	19	2971143.708			

**Table 4:** Regression intercept and slope

	Coefficients	Standard error	t stat	P-value	Lower 95%	Upper 95%
Intercept	255.8196191	95.70261635	2.673068186	0.015512959	54.75588306	456.8833551
Slope	0.247080364	0.183267973	1.348191718	0.194318836	-0.137951361	0.632112088

**Figure 3:**
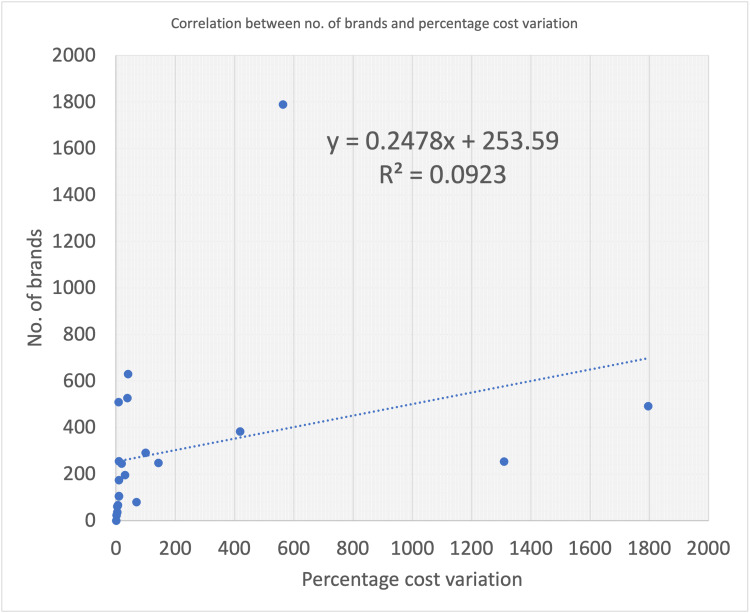
Correlation between the number of brands and percentage cost variation

Figure 3 shows the graphical representation of the correlation between the number of brands and percentage cost variation.

The data show a 9% positive correlation between the increase in the number of brands with percentage cost variation. The Spearman correlation constant from the data came out to be 0.303.

## Discussion

Health economics has always played a pivotal role in deciding the choice of drug for a particular indication. The cost of drugs modifies the choices made at all levels, from the stage of formulary management in the hospital to a physician deciding on a drug for a particular patient based on his financial condition and the indication to ensure better compliance and complete recovery. It is important to ensure that drugs are priced correctly to ensure that patients do not face a lot of burden because of medicines [7,8], and also ensure that different brands for a particular combination of drugs do not differ much in price so that the patients can choose between the brands based on their availability if the efficacy and bioavailability remain the same [9].

In our study, we found that out of 21 combinations listed in this study, only seven had a favorable cost ratio of <2, and five had a favorable percentage cost variation of less than 100. This is not a positive finding as less than 1/3rd of the combinations are priced in a manner that will not have an economic burden on patients, a deterrent for them to continue medication and complete therapy. The minimum cost ratio (1.22) and percentage cost variation (22.80%) is for the combination of pantoprazole 40 mg + domperidone (immediate release (IR)) 10 mg + domperidone (sustained release (SR)) 20 mg. A possible reason for this is that there are only two brands available for this combination in the market.

The highest cost ratio (18.88) and percentage cost variation (1788.88%) have been reported for a combination of rabeprazole 20 mg and domperidone 10 mg. Even though there are 563 brands available in the market selling this combination, the possible reason for this price variation could be the fact that rabeprazole has the fastest onset of action as compared to other PPI drugs [10]. Pharmaceutical companies could leverage this to increase the cost of the drug. Another study conducted by Bhave et al. also showed that the highest cost ratio and percentage cost variation was for rabeprazole 20 mg + domperidone 30 mg [11], owing to the same reason as above. The second highest percentage cost variation (629.09%) in our study was for pantoprazole 40 mg and itopride 150 mg, possibly because of fewer brands (40) selling this particular combination.

We also found that the combination of pantoprazole 40 mg and domperidone 30 mg has the most number of brands available in the market (1796), followed by pantoprazole 40 mg and domperidone 10 mg (1310). This could be because of the widespread use of this combination that motivates manufacturers to make more bio-equivalent brands of the same [12]. Even though there is competition between the brands, the cost ratio and percentage cost variation are not favorable. Most of the combinations that show a favorable ratio is because fewer brands are available in the market. Rabeprazole 20 mg + domperidone (IR) 10 mg + domperidone (SR) 20 mg has only one brand available in the market so analysis of the cost for the same could not be done.

From the correlation statistics, we found that for each increase in the number of brands, the percentage cost variation increases by 0.24% (p-value: 0.194). This is not a significant value, but it shows that an increase in sales of a particular combination does not bring down the price as we would expect it to, but rather it allows companies to set higher prices.

We have taken cost information only from two sources, so we might have missed the brands that are not mentioned in them. Also, this study evaluates only the variation in the price of drugs but further studies can be done that take the efficacy into perspective.

From the study, we found that there is a wide variation in the price of drugs between different brands of a particular combination. This can lead to a lot of economic burden on the patients. According to the National Sample Survey data, health-related expenditures can push around 20 million people below the poverty line every year in India [13]. It is imperative to bring about changes in the policy so that the cost price of drugs is not variable to the extent that patients find it difficult to afford and complete their therapy. Stricter monitoring by the government can bring about a positive change in this direction. Physicians can also be made aware of the prices of drugs so that they prescribe the best available alternative to the patients, as the efficacy of most PPIs remains the same.

## Conclusions

We found a wide variation in price amongst different brands of various combinations of PPIs. Very few combinations have a favorable difference in price. This could lead to an increase in the financial burden on patients and substantially decrease the compliance of the patients to therapy. Physicians need to be made aware of these price differences so that they can choose the best available alternative for each patient.
